# Cardioprotective Effects of Glycyrrhizic Acid Against Isoproterenol-Induced Myocardial Ischemia in Rats

**DOI:** 10.3390/ijms12107100

**Published:** 2011-10-21

**Authors:** Nagaraja Haleagrahara, Julian Varkkey, Srikumar Chakravarthi

**Affiliations:** 1 Human Biology Division, School of Medicine, International Medical University, Kuala Lumpur 57000, Malaysia; 2 Division of Postgraduate Studies, International Medical University, Kuala Lumpur 57000, Malaysia; E-Mail: julian-v@live.com; 3 Pathology Division, School of Medicine, International Medical University, Kuala Lumpur 57000, Malaysia; E-Mail: srikumar_chakravarthi@imu.edu.my

**Keywords:** oxidative stress, isoproterenol, glycyrrhizic acid, lipid hydroperoxides, 8-isoprostane

## Abstract

The aim of the present study was to look into the possible protective effects of glycyrrhizic acid (GA) against isoproterenol-induced acute myocardial infarction in Sprague-Dawley rats. The effect of three doses of glycyrrhizic acid in response to isoproterenol (ISO)-induced changes in 8-isoprostane, lipid hydroperoxides, super oxide dismutase and total glutathione were evaluated. Male Sprague-Dawley rats were divided into control, ISO-control, glycyrrhizic acid alone (in three doses-5, 10 and 20 mg/kg BW) and ISO with glycyrrhizic acid (in three doses) groups. ISO was administered at 85 mg/kg BW at two consecutive days and glycyrrhizic acid was administered intraperitoneally for 14 days. There was a significant increase in 8-isoprostane (IP) and lipid hydroperoxide (LPO) level in ISO-control group. A significant decrease in total superoxide dismutase (SOD) and total glutathione (GSH) was seen with ISO-induced acute myocardial infarction. Treatment with GA significantly increased SOD and GSH levels and decreased myocardial LPO and IP levels. Histopathologically, severe myocardial necrosis and nuclear pyknosis and hypertrophy were seen in ISO-control group, which was significantly reduced with GA treatment. Gycyrrhizic acid treatment proved to be effective against isoproterenol-induced acute myocardial infarction in rats and GA acts as a powerful antioxidant and reduces the myocardial lipid hydroperoxide and 8-isoprostane level.

## 1. Introduction

Myocardial infarction (MI) occurs when there is myocardial necrosis due to prolonged imbalance between the myocardial oxygen supply and demand of the myocardium [1]. Myocardial infarction is said to be part of a spectrum of diseases known as Acute Coronary Syndromes (ACS). The diseases that make up the spectrum are unstable angina, acute myocardial infarction, and sudden cardiac death [2,3]. Among the various proposed mechanisms, the accumulations of free radicals have been implicated in the pathophysiology of acute myocardial infarction [4]. Isoproterenol is a beta-adrenoceptor agonist that induces myocardial infarction by causing imbalance between oxidants and antioxidants in the myocardium [5,6].

Several antioxidants have been tested for their possible protective actions against acute myocardial infarction [7,8]. Antioxidants suppress the formation of reactive oxygen species and shift the balance towards antioxidants from pro-oxidants, which accumulate to protect the myocytes [6,9]. Glycyrrhizic acid (GA) is a triterpenesaponin glycoside, which is the primary bioactive component of the main extract of the root of the plant GlyccyrhizaGlabra (Liquorice), a shrub from the Leguminosae family [10,11]. Research has shown that glycyrrhizic acid exhibits anti-ulcerative, expectorant, anti-viral, anti-inflammatory, anti-diabetic, anti-cancer, neuroprotective, and immune enhancing properties [10,12,13]. A recent report by Lee *et al*. [11] demonstrated that glycyrrhizic acid was also able to attenuate oxidative damage induced by carbon tetrachloride induced hepatic damage in mice. Another report [14] shows that glycyrrhizic acid is able to reduce oxidative stress damage in ischemia reperfusion injuries.

In view of the protective role of antioxidants against isoproterenol-induced myocardial infarction, we have taken up the present research to evaluate the cardioprotective effect of glycyrrhizic acid against acute myocardial ischemia. Molecular mechanism of glycyrrhizic acid was studied through biochemical and histopathological approach in this study. We hypothesized that glycyrrhizic acid has a cardioprotective effect against isoproterenol-induced myocardial damage.

## 2. Results and Discussion

### 2.1. Body Weight and Heart Weight

There was a significant difference in the body weight between control and ISO-control group (*p* < 0.05). Isoproterenol treatment significantly decreased the body weight of the rats, compared to control and GA alone groups (*p* < 0.05). Treatment with GA for ISO groups significantly increased (*p* < 0.05) the body weight and GA-20 mg dose was able to increase the body weight to near normal control level. ISO treatment significantly increased (*p* < 0.05) the heart weight showing ISO-induced cardiac hypertrophy in rats. GA treatment to ISO groups significantly reduced the heart weight (*p* < 0.05) than ISO-control group, but GA-5 mg group had higher heart weight than control rats. A higher dose of GA significantly reduced (*p* < 0.05) cardiac hypertrophy caused by isoproterenol treatment (Table 1).

### 2.2. Cardiac Marker Enzymes

There was a significant increase in mean CK-MB levels in the ISO alone group when compared to the control group (*p* < 0.05). When the control group was compared with ISO groups treated with all three doses of GA, a significant increase in mean CK-MB levels was also seen (*p* < 0.05). There was no significant difference between ISO groups treated with 5 mg GA, 10 mg GA, and 20 mg GA (*p* > 0.05). When compared between ISO alone group with ISO groups treated with all three doses of GA, a significant reduction of CK-MB levels was seen (*p* < 0.05). During pair-wise comparison, a significant increase of mean LDH levels was found in the SO-control group when compared to the control group (*p* < 0.05). ISO with 5, 10 and 20 mg GA, also showed a significantly higher level of LDH when compared to the control group (*p* < 0.05). There was no significant difference between LDH levels with GA alone treatment at different doses (Table 2).

### 2.3. Lipid Hydroperoxides

**Figure 1:**
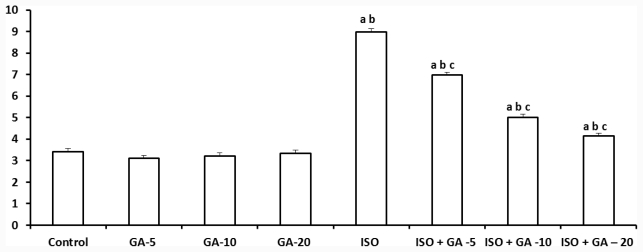
Effect of glycyrrhizic acid on tissue lipid hydroperoxide (LPO) level (nmol/mg protein) in isoproterenol-induced rats. Values are given as means ± S.D. for six rats. a: Significantly different from control (*p* < 0.05); b: Significantly different from glycyrrhizic acid (GA) alone treatment group (*p* < 0.05); c: Significantly different from Iso-control group (*p* < 0.05).

When compared with the pair-wise technique, the ISO-control group showed a significant increase (*p* < 0.05) in cardiac LPO levels compared to the control group. GA alone treated groups at all three doses did not show any significant change in LPO level. GA treatment to ISO groups resulted in significant decrease (*p* < 0.05) in LPO level, compared to the ISO-control group. GA at all three doses decreased LPO levels significantly, but the level was significantly higher (*p* < 0.05) than control rats (Figure 1).

### 2.4. 8-Isoprostane

**Figure 2:**
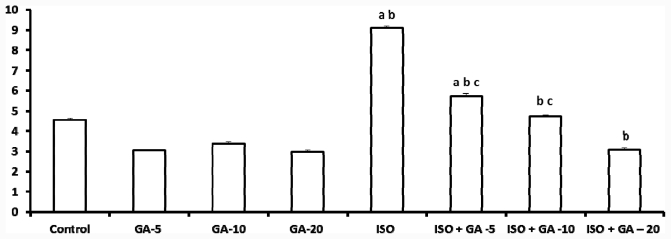
Effect of glycyrrhizic acid on tissue 8-isoprostane level (pg/mL/mg protein) in isoproterenol-induced rats. Values are given as means ± S.D. for six rats. a: Significantly different from control (*p* < 0.05); b: Significantly different from GA alone treatment group (*p* < 0.05), c: Significantly different from Iso-control group (*p* < 0.05).

Treatment with isoproterenol produced a significant increase in 8-Isoprostane (IP) as compared to the control group (*p* < 0.05). There was no significant difference seen between non-ISO groups treated with all three doses of GA, when compared to the control group (*p* > 0.05). Upon induction of MI with isoproterenol, the ISO-control group showed significantly higher IP levels as compared to the GA alone groups and control group (*p* < 0.05). When ISO groups were treated with all three doses of GA, levels of IP dropped to levels significantly lower than those of ISO-alone group (*p* < 0.05). In ISO + 5 mg GA, the level dropped, but only to a level that still showed difference which was significantly higher than the control group (*p* > 0.05). For ISO-10 mg GA and ISO-20 mg GA however, levels of IP dropped even further to the point that they were closer to that of control group (Figure 2).

### 2.5. Superoxide Dismutase

**Figure 3:**
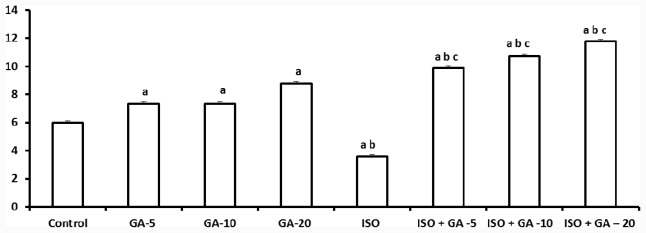
Effect of glycyrrhizic acid on tissue total SOD level (U/mg protein) in isoproterenol-induced rats. Values are given as means ± S.D. for six rats. a: Significantly different from control (*p* < 0.05); b: Significantly different from GA alone treatment group (*p* < 0.05); c: Significantly different from Iso-control group (*p* < 0.05).

Total SOD decreased significantly after isoproterenol treatment (*p* < 0.05), but treatment with GA significantly increased the SOD levels after 14 days (*p* < 0.05). ISO with GA-5, 10 and 20 mg/kg, increased the SOD levels more than ISO-control group (*p* < 0.05) and this level was significantly higher than the control group (*p* < 0.05). GA alone at 20 mg/kg at three doses significantly increased (*p* < 0.05) total SOD level, more than control group (Figure 3).

### 2.6. Total Glutathione

**Figure 4:**
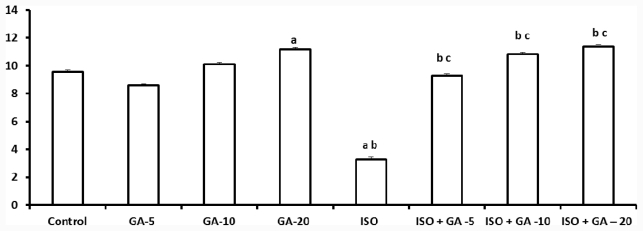
Effect of glycyrrhizic acid on tissue total glutathione (GSH) level (nmol/mg protein) in isoproterenol-induced rats. Values are given as means ± S.D. for six rats. a: Significantly different from control (*p* < 0.05); b: Significantly different from GA alone treatment group (*p* < 0.05); c: Significantly different from Iso-control group (*p* < 0.05).

The isoproterenol alone group showed a significant fall in the mean level of GSH when compared to the control group (*p* < 0.05). Following administration of 20 mg/kg body weight GA to non-ISO groups, mean levels of GSH increased significantly when compared to control group and ISO alone group (*p* < 0.05). In contrast, the ISO-control group showed a significant decrease in mean GSH levels when compared to control group (*p* < 0.05). This was reversed upon treatment with all three doses of GA, causing levels of GSH to rise to be significantly higher than the MI alone group (*p* < 0.05). ISO + 5 mg GA rose to a level more or less similar to that of the control group with statistical test showing no significant difference between them (*p* > 0.05). ISO + 10 mg GA and ISO + 20 mg GA showed significantly higher GSH than those of the control group (*p* < 0.05) (Figure 4).

### 2.7. Histopathological Analyses

**Figure 5:**
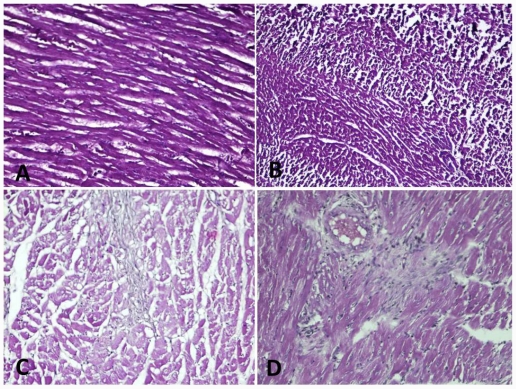
Histopathological analysis of rat myocardium: (**A**) Normal appearance myocardial tissue in control rats. (**B**) Normal myocardium without any necrosis or nuclear pyknosis in GA alone group. (**C**) Myocardial infarction with ISO treatment showing necrosis and scar formation. (**D**) Vascular proliferation and macrophage activity in ISO + GA treated group (200×).

The myocardium showed adequate cellularity and normal morphology. Myocytes were healthy and there was no evidence of myocyte necrosis, nuclear pyknosis, vascular proliferation, macrophage activity, scar formation, or muscle hypertrophy. Treatment with glycyrrhizic acid alone at three doses showed that the myocardium was similar to that of the control group with adequate cellularity and normal morphology. No evidence of necrosis, nuclear pyknosis, angiogenesis, vascular proliferation, macrophage activity, scar formation, or muscle hypertrophy was seen (Table 3, Figure 5). In the ISO-control group there were morphological changes that were strongly suggestive of isoproterenol-induced myocardial injury were seen. Large areas of coagulative necrosis were seen with neutrophilic infiltrate, diffused interstitial edema and pale myocytes with fading nuclei and decreased striations. Nuclear pyknosis and clumping of cytoplasm was evident throughout areas of necrosis. In ISO groups treated with GA, many areas of myocyte debris were disintegrated with the presence of macrophages, suggesting that the myocytes were removed by macrophage activity. Macrophage activity was prominent in areas of injury and it increased in order of the increasing doses of GA. This was also true for areas of scar formation, and areas of vascular proliferation which were both present in numerous areas throughout the myocardium and increased in order of the increasing doses of GA. Increased areas of scar formation, vascular proliferation and macrophage activity were indicative of better healing following myocardial infarction (Table 3, Figure 5).

## 3. Discussion

The results of the present study showed that there was significant increase in oxidative stress after myocardial ischemia in rats and glycyrrhizic acid showed a significant protective effect against this oxidative damage. There was no significant difference in the effect of different dose of glycyrrhizic acid in majority of the parameters studied.

There was a significant elevation in serum lactate dehydrogenase (LDH) and creatine kinase-MB (CK-MB) confirming the acute myocardial infarction in rats. These observations are in line with previous studies done on rats treated with isoproterenol [15,16]. The myocardial cells contain many cardiac enzymes like creatinine kinase, lactate dehydrogenase, asparate transaminase *etc*. Upon administration of isoproterenol, the oxygen demand of the heart increases with increase in ionotropic effect in the heart, resulting in prolonged ischemia and glucose deprivation. The cells are damaged with increased muscle contractility, which results in increasing the cell membranes permeability allowing cardiac enzymes to leak out into the bloodstream [9,15–18]. Creatine kinaseis an enzyme capable of reversibly transferring a phosphate group from the energy storage form of creatine phosphate, to a molecule of ADP, producing ATP [19]. CK-MB is localized predominantly in the heart and this makes it a valuable diagnostic tool for MI since damage specific to the myocardium would result in elevation of CK-MB levels [19–21]. CK- MB estimation is considered the standard to which all cardiac biomarkers will be compared to [19,22]. LDH has been used traditionally as a nonspecific diagnostic tool for myocardial infarction. A rise in the proportion of LDH in the serum can be diagnostic of myocardial infarction [23,24]. LDH usually rises within 6–12 hours of MI. Level of LDH peaks within 48 hours, remains at that peak for 4–14 days. Histopathological study also confirmed the myocardial damage with ISO treatment. There were nuclear pyknosis and necrosis in the myocardium. Vascular proliferation, interstitial edema, increased macrophage activity, scar formation, and myocardial hypertrophy confirmed the myocardial infarction with isoproterenol treatment [15,16,25].

An increase in oxidative stress was recorded during and following myocardial infarction by many researchers [26–29]. This may cause oxidative damage to proteins and lipids interfering with myocyte structure and functions. The observed increasein LPO with isoproterenol could be because of formation of free radicals and also through exhaustion of antioxidants, leading to oxidative stress. Stimulation of beta adrenergic receptors with isoproterenol treatment generates excess reactive oxygen species in myocardium [9,16]. Reactive oxygen species (ROS) attack polyunsaturated fatty acids, which is the precursor of lipid peroxide formation [30]. Increased myocardial LPO is suggestive of lipid accumulation and irreversible myocardial damage. Intense lipid peroxidation caused by isoproterenol may affect the mitochondrial and cytoplasmic membrane causing more severe oxidative damage in the heart and consequently releasing LPO into circulation [30–32].

Although isoprostanes can be produced by cyclooxygenase enzymes *in vitro* and *in vivo*, its production *in vivo* is almost entirely attributed to oxidation of lipids due to reactive oxygen species [33]. In fact, the majority of IPformation happens in the esterified arachidonic acids in membrane phospholipids. These IP are then released into the circulation by the action of phospholipases [34]. Aside from just being markers of lipid peroxidation, isoprostanes have also been shown to possess biological activity and are capable of mediating oxidative stress. Increased 8-isoprostane in this study with ISO confirms the oxidative stress in myocardium. Isoprostane has vasoconstriction and platelet aggregation property which might have aggravated the myocardial injury with ISO treatment [35,36].

Increase in oxidative stress with ISO treatment causes oxidation of lipids, proteins and DNA in the myocardium causing alteration in cell structure and function. This eventually leads to myocardial injury. The ISO alone group showed a significant decrease in myocardial endogenous antioxidants SOD and GSH when compared to control and non-MI groups. Isoproterenol is a synthetic catecholamine and catecholamines rapidly undergo oxidation and oxidation products of metabolism will have significant effect on myocardium. SOD, and GSH take part in maintaining glutathione homeostasis in the tissues. These antioxidants are involved in the defense system against free radical mediated tissue or cellular damage [15,16,18,21].

GSH is ubiquitous antioxidant, and essential biofactor synthesized in all living cells. It protects the cells from free radical mediated injury caused by drugs. It forms an important substrate for several other antioxidant enzymes. SOD activity decreased significantly with isoproterenol due to an excessive formation of superoxide anions. A decrease in SOD activity may result in the decreased removal of superoxide anions, which can cause free radical-induced myocardial damage [37,38]. Decrease in the level of SOD and GSH in myocardium is in close relationship with increased LPO and 8-IP. SOD and GSH are the primary defense antioxidants, the level of which depleted significantly with isoproterenol-induced myocardial ischemia suggestive of severe oxidative stress in the heart.

Treatment with glycyrrhizic acid decreased LPO and IP levels and increased SOD and GSH level in the heart. This supports the scavenging activities of GA and protecting the myocardial antioxiodant enzymes. A recent study [11] showed that GA was capable of reducing oxidative stress and lipid peroxidation caused by carbon tetrachloride induced liver injury in mice [11,14]. Other studies have been recorded that also state the antioxidant properties of GA [14,39]. GA’s antioxidant properties could be due to the fact that it is able to achieve maximum conjugation with free radicals, thus neutralizing them [11]. GA is also capable of reducing the production of free radicals by neutrophils and interfering with neutrophil chemotaxis [10,14]. Considering neutrophils play a major role in the pathogenesis of acute myocardial infarction, inhibition of their ROS formation will significantly reduce oxidative stress during myocardial infarction. GA’s ability to neutralize free radicals and to interfere with neutrophil chemotaxis and ROS formation makes it a useful antioxidant for reducing oxidative stress during infarction. This confirms that GA is a good cardioprotective agent. Apart from this, GA administration in non-ISO groups caused an increase in endogenous antioxidants SOD and GSH as well which suggest that GA may be able to independently increase the pool of GSH and SOD in the myocardium without the presence of oxidative stress and neutrophils. This confirms that the antioxidant effects of GA increase endogenous antioxidant enzymes even in normal cardiac tissue. Relatively very few data are available about the antioxidant properties of glycyrrhizic acid. In fact, this is the first study reporting the antioxidant and cardioprotective effects of glycyrrhizic acid in isoproterenol-induced myocardial ischemia in rats. When the effects of three different doses of GA were compared, it was observed that the higher dose of GA (10 and 20 mg/kg body weight) had a significant effect than the lower dose (5 mg/kg body weight).

Histopathological findings of isoproterenol treated rats showed infarction zone with myocardial necrosis, nuclear pyknosis, and hypertrophy and scar formation. Glycyrrhizic acid treatment to ISO groups had shown a significant protection against myocardial injury. Different dose of GA has shown that many areas of myocyte debris were disintegrated with accumulation of macrophages, suggesting that the myocytes were removed by these macrophages. Myocardial healing was seen with different dose of GA in ISO treated rats suggesting protective function of GA in myocardial infarction. Treatment with GA alone at three doses did not show any necrosis or scar formation or hypertrophy. This indicates that glycyrrhizic acid does not possess any adverse effects to myocardium under normal conditions.

## 4. Experimental Section

Adult, male Sprague-Dawley rats (200–225 g) were used for this study. Rats were allowed to adapt to the laboratory conditions at least one week before the start of the experiments. The rats were placed in standard laboratory conditions on arrival in an air-conditioned room (25 ± 2 °C) with controlled 12 h light/12 h dark cycles. Rats were placed in polypropylene cages (3 per cage). Food and water were available freely all the time except during the experimental procedures. All the experimental procedures were carried out according to the Guidelines for Ethical care and Use of Experimental Animals and the study protocol was approved by Institutional Research and Ethics Committee.

Isoproterenol hydrochloride and glycyrrhizic acid were purchased from Sigma-Aldrich. The rats were randomly assigned into eight groups (six rats per group). Group 1, control; group 2, 3 and 4 glycyrrhizic acid (GA) alone in three different doses (5, 10 and 20 mg/kg BW); group 5, ISO-control group; group 6, 7 and 8, ISO with GA (5, 10 and 20 mg/kg BW). Isoproterenol was administered subcutaneously (85 mg/kg BW) for two days. GA was administered intraperitoneally after 48 hours to all the rats in GA alone and ISO + GLY groups at three doses (5 mg/kg, 10 mg/kg and 20 mg/kg BW) for 14 days.

Twenty-four hours after the last treatment, the rats were weighed, and then sacrificed with over dose of anesthesia (50 mg/kg BW of sodium pentobarbitol) and blood samples were collected by cardiac puncture. The heart was dissected and weighed. Blood was centrifuged and serum was separated from which cardiac biomarkers, creatinine kinase-MB (CK-MB) and lactate dehydrogenase were estimated spectrophotometrically using commercially available kits (Bioassay Systems, CA, USA). Part of the heart was dissected and homogenized immediately and supernatant was kept at −80 °C until further biochemical assay. From the homogenate samples, lipid hydroperoxides (LPO), 8-isoprostane level (IP), total superoxide dismutase (SOD), total glutathione (GSH) and total protein levels were assayed using ELISA kits (Cayman Chemicals and Pierce Biotechnology, USA). Tissue LPO, IP, SOD and GSH levels were expressed per milligrams of proteins. Another portion of the heart was placed in formalin (10%). Paraffin blocks were prepared from the heart and thin sections were cut (4 μM) and stained with haematoxylin-eosin (H&E) using the staining protocol for light microscopic examination. The sections were evaluated under light microscope (200×) for necrosis, nuclear pyknosis, hypertrophy, angiogenesis, scar formation and macrophage activity. Severity of changes were graded using a scale of no change (−), mild changes (+), moderate changes (++) and severe changes (+++).

### 4.1. Lipid Hydroperoxide (LPO) Assay

A quantitative extraction method as provided in the ELISA kit method for LPO assay was used to extract lipid hydroperoxides into chloroform and the extract was directly used. This procedure eliminates any interference caused by hydrogen peroxide or endogenous ferric ions in the sample and provides a sensitive and reliable assay for lipid peroxidation. The absorbance was read at 500 nm using 96 well plate reader and a dose response curve of the absorbance unit versus concentration in nano moles was prepared. LPO levels were expressed as nmol/mg of protein.

### 4.2. 8-Isoprostane (IP) Assay

8-isoprostane assay is based on competition between 8-Iso and an 8-Iso-Acetylcholinesterase conjugate (tracer) for a limited number of rabbit antiserum sites. During the assay, the concentration of tracer was held constant while the concentration of 8-Iso varied according to sample. Therefore, amount of tracer able to bind with serum was inversely proportional to the concentration of 8-Iso. This rabbit antiserum complex then bound to the rabbit IgG antibodies previously attached to the wells. Plates are then washed to remove unbound reagents and Ellman’s reagent (contains tracer substrate) was added. The yellow mixture produced was quantified spectrophotometrically at absorbance of 412 nm. IP were expressed as pg/mL/mg of protein.

### 4.3. Superoxide Dismutase (SOD) Assay

This assay kit utilizes a tetrazolium salt for the detection of total superoxide radicals (O^2−^) generated by xanthine oxidase and hypoxanthine. One unit of SOD is defined as the amount of enzyme necessary to exhibit 50% dismutation of superoxide radical. Oxidation rate of tetrazolium salt to formazan dye by O^2−^ is inversely proportional to the endogenous activity of SOD. The formazan dye stains the 96 wells and its staining intensity was detected by absorbance spectrophotometry at 450 nm using a plate reader. Total SOD levels were determined from a standard curve and expressed as U/mg of protein.

### 4.4. Total Glutathione (GSH) Assay

Glutathione assay kit utilizes an optimized enzymatic recycling method that uses glutathione reductase for quantification of GSH. GSH exerts its antioxidant action through its sulfhydryl groups. This assay takes advantage of the sulhydryl groups reaction with DTNB (5,5′-dithio-bis-2-nitrobenzoic acid) producing a yellow coloured 5-thio-2-nitrobenzoic acid (TNB). The mixed disulfide of TNB and GSH produced is further reduced by glutathione reductase to recycle the GSH and produce more GSH. Rate of TNB production is directly proportional to the recycling reaction and the concentration of GSH in the tissue sample. TNB was then quantified spectrophotometrically at an absorbance of 405 nm. GSH was expressed as nmol/mg protein.

### 4.5. Statistical Analysis

All the results were reported as means with standard deviation. Graph Pad prism 5.0 software used for statistical analyses. Comparison between different groups were done using Kruskal Wallis one way analysis of variance test. Pair wise comparison between the different groups was done using Mann-Whitney-U test. *p* < 0.05 was considered to show statistical significance.

## 5. Conclusions

Histopathological and biochemical findings of the present study indicate that glycyrrhizic acid possess antioxidant properties in myocardium and protects against isoproterenol-induced oxidative stress. The most important protective mechanism offered by glycyrrhizic acid is through its ability to decrease lipid hydroperoxides and isoprostanes and to increase the superoxide dismutase and glutathione level. Thus, glycyrrhizic acid has been shown to possess cardioprotective effect against isoproterenol-induced acute myocardial infarction in rats.

## Figures and Tables

**Table 1:** Effect of glycyrrhizic acid on body weight and heart weight in isoproterenol-induced rats.

Groups	Body Weight (g)	Heart Weight (g)
**Control**	202.43 ± 4.47	0.263 ± 0.021
**GA-5**	196.12 ± 3.21 a	0.289 ± 0.029
**GA-10**	205.34 ± 4.85	0.291 ± 0.020 a
**GA-20**	210.09 ± 5.21 a	0.267 ± 0.031
**ISO-Control**	183.56 ± 3.64 a	0.398 ± 0.031 ab
**ISO + GA-5**	190.34 ± 4.21 ab	0.306 ± 0.008 ab
**ISO + GA-10**	195.87 ± 3.20 c	0.287 ± 0.012 c
**ISO + GA-20**	198.34 ± 2.22 c	0.280 ± 0.020 c

Values are given as means ± S.D. for six rats.

a Significantly different from control (*p* < 0.05);

b Significantly different from GA alone treatment group (*p* < 0.05);

c Significantly different from Iso-control group (*p* < 0.05).

**Table 2:** Effect of glycyrrhizic acid on serum cardiac markers in isoproterenol-induced rats.

Groups	CK-MB (U/L)	LDH (mmol/L)
**Control**	3.763 ± 0.371	20.318 ± 1.407
**GA-5**	3.601 ± 0.238	18.942 ± 0.751
**GA-10**	3.669 ± 0.412	20.308 ± 1.581
**GA-20**	3.593 ± 0.623	20.319 ± 0.845
**ISO-Control**	10.702 ± 0.741^a^^b^	72.224 ± 2.641^a^^b^
**ISO + GA-5**	7.562 ± 0.817 abc	39.283 ± 2.794^a^^b^^c^
**ISO + GA-10**	6.372 ± 0.552 abc	34.789 ± 2.981 abc
**ISO + GA-20**	6.455 ± 0.848 abc	37.617 ± 3.423 abc

Values are given as means ± S.D. for six rats.

a Significantly different from control (*p* < 0.05);

b Significantly different from GA alone treatment group (*p* < 0.05);

c Significantly different from Iso-control group (*p* < 0.05).

**Table 3:** Effect of glycyrrhizic acid on histopathological changes in the hearts of isoproterenol-induced rats.

Groups	Necrosis	Pyknosis	Angiogenesis	Scar Formation	Hypertrophy	Macrophage Activity
**Control**	−	−	−	−	−	−
**GA-5**	−	−	−	−	−	−
**GA-10**	−	−	−	−	−	−
**GA-20**	−	−	−	−	−	−
**ISO-Control**	+++	++	++	+	++	++
**ISO + GA-5**	++	++	++	+	+	+
**ISO + GA-10**	+	+	++	+	+	+
**ISO + GA-20**	+	+	+++	++	+	+

Histopathological changes; −No change, + Mild, ++ Moderate, +++ Severe.
